# Urban Low-Rise Residential Areas Provide Preferred Song Post Sites for a Resident Songbird

**DOI:** 10.3390/ani12182436

**Published:** 2022-09-15

**Authors:** Yanhong Chen, Lijing Li, Xiaotian Zhu, Yicheng Shen, Anran Ma, Xinyu Zhang, Pan Chen, Changhu Lu

**Affiliations:** 1 Anhui Provincial Key Laboratory of the Conservation and Exploitation of Biological Resources, College of Life Sciences, Anhui Normal University, Wuhu 241000, China; 2 School of Ecology and Environment, Anhui Normal University, Wuhu 241000, China; 3 College of Biology and the Environment, Nanjing Forestry University, Nanjing 210037, China

**Keywords:** oriental magpie-robin, urbanization, bird song, high-rise building, urban ecology

## Abstract

**Simple Summary:**

Songbirds adjust their song traits to adapt to urban environments from rural ones. However, the effects of the intraurban environmental variation on resident birds have received little attention. Here, we assessed the distribution and song differences of a common songbird—the oriental magpie-robin—between three urban habitat types. Population density and song diversity were higher in low-rise residential areas than in urban parks, while high-rise residences were rejected by birds. Overall, these results suggest that low-rise residential areas may provide preferred song post sites for this urban resident bird.

**Abstract:**

Urbanization is expanding rapidly worldwide, and brings additional selection pressure on animals. The song differences between urban and rural songbirds have been widely verified, but the effects of urban morphological variation on long-settled urban birds have been poorly explored. Here, we investigated the distribution and song differences of a common resident songbird—the oriental magpie-robin (*Copsychus saularis*) between three urban morphology types (i.e., urban park, low-rise residential area, and high-rise residential area). The results indicated that the population density in low-rise residential areas was significantly higher than in urban parks, while it was the lowest in high-rise residential areas. Males in low-rise residential areas had greater song length, syllable numbers, frequency bandwidth, and song diversity than those in urban parks. The song differences were mainly related to habitat types, independent of singing height and perch type. Our findings suggest that low-rise residential areas may provide preferred song post sites for the oriental magpie-robin, which is well-adapted to the low-rise building morphology, but rejects the emerging high-rise buildings. Future studies are needed to assess the effects of urban morphological variation on more resident animals to determine which urban morphologies are conducive to enhancing biodiversity and encouraging animals to settle in urban areas.

## 1. Introduction

In recent decades, urban environments have expanded rapidly worldwide, and urbanization constantly brings new selection pressure, with enormous effects on animals [1,2]. Birds are one of the most widely distributed groups of terrestrial vertebrates, occupying a variety of habitat types in modern ecosystems [3]. A series of environmental changes caused by urbanization affect the evolutionary process of avian biodiversity, including habitat fragmentation [4], vegetation homogenization [5], the heat island effect [6], and increases in light and noise [7,8]. These phenomena obviously lead to severe challenges in biodiversity conservation. However, in some respects, cities are also ideal laboratories for studying natural selection. Some species have adapted to the new pressures in urban environments, where they coexist near humans, providing a unique perspective for exploring evolutionary and selective processes [9].

Many studies on the adaptation and evolution of urban birds suggest that the behavior of birds is largely limited by environmental factors, but that they also have the plasticity to adapt to the urbanization process [10,11]. A typical study is the effect of urban noise on the variation of birdsong, e.g., in high-noise urban areas, great tits (*Parus major*) increase their song frequency to overcome the masking effect of noise [12]. Meanwhile, a variety of other factors associated with urbanization may also drive changes in birdsong, e.g., great tits are capable of adjusting their songs to changes in vegetation cover and diversity in urban green spaces [13]; saffron finches (*Sicalis flaveola*) living in highly urbanized sites sing earlier at dawn to cope with artificial light at night [8]; the active space of the brown-headed cowbird (*Molothrus ater*)’s song is affected by both habitat type and level of urbanization [14]. However, most previous studies have focused on the comparison between urban and rural areas in terms of birdsong. Environmental differences within cities also affect birds’ community structure and distribution, e.g., the differences in the urban morphology (i.e., building height and density) in parks or residential areas [15]. With the development of urbanization in China, more and more high-rise residential buildings (at least 10–15 stories, with many even over 30 stories) have been constructed in recent years [16]. It is not clear whether the variation in urban morphology may affect the habitat selection of long-settled urban birds, as well as their songs.

The oriental magpie-robin (*Copsychus saularis*) is a common small songbird with a natural geographic distribution spanning south of the Yangtze River in China through most of South and South-East Asia, and it can be found in a variety of open habitats in urban and rural areas [17]. This bird is often a resident close to human habitation, and has a limited range of activities because it is poor at long-distance flight. In the breeding season, males have very diverse and complex songs for establishing territories [18,19]. The loud vocals and flexible song characteristics of the oriental magpie-robin make it an ideal species for examining the effects of urban environmental factors on avian acoustic communication [20,21]. In this study, we investigated the differences in the population density of the oriental magpie-robin between different habitat types (i.e., urban parks, low-rise residential areas, and high-rise residential areas) in the urban environment, the differences in song characteristics between urban parks and residential areas, and the key environmental variables predicting song differences. Our study may provide new insights for better understanding of the population distribution and song variation of urban resident songbirds in the process of adapting to rapid urban change.

## 2. Materials and Methods

### 2.1. Study Site

This work was conducted in the urban area of the city of Wuhu (30°38′ N–31°34′ N, 117°57′ E–118°54′ E) in Anhui Province, China. The region is dominated by plains and hills, located on the south bank of the middle–lower reaches of the Yangtze River, with a subtropical humid monsoon climate (the annual average temperature is 15.7–16.0 °C, and the average precipitation is 1198.1–1413.2 mm). The urban area maintains several parks with semi-natural vegetation, with high greening and a variety of habitat types, providing suitable habitats for birds. With the accelerated population growth and urbanization, as in many other Chinese cities, high-rise residential areas (about half greater than 30 stories) have gradually increased and rapidly replaced low-rise residential areas (no higher than 6 stories) in Wuhu in the last decade. The oriental magpie-robin is a common resident bird in the urban area of Wuhu, which is often active in parks and residential areas.

### 2.2. Field Sampling

**Figure 1:**
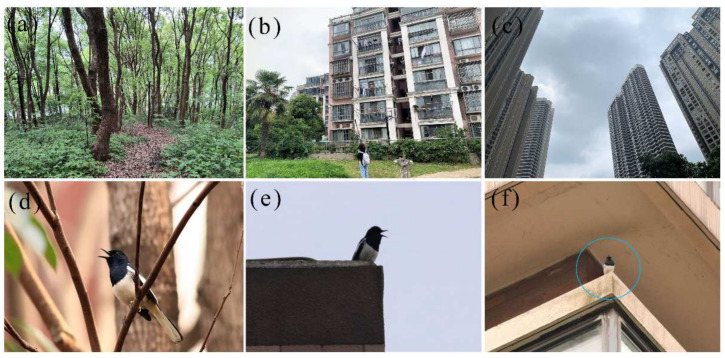
The three habitat types ((**a**) urban park; (**b**) low-rise residential area; and (**c**) high-rise residential area) investigated in the urban area of the city of Wuhu, and individual oriental magpie-robins singing in different habitats ((**d**) in a tree in an urban park; (**e**) on the roof of a low-rise residential area; and (**f**) in the middle of the floor of a high-rise residential area).

To examine the distribution of oriental magpie-robins in the urban area, we selected three types of habitat (i.e., urban park, low-rise residential area, and high-rise residential area, Figure 1a–c) for the survey of population density. All selected urban parks were more than 10 years old, located in the urban core, with a greening rate of about 80–90% and a few low-rise buildings. The height of the buildings in low-rise residential areas was no higher than 6 stories, while those in high-rise residential areas were higher than 30 stories; all residential areas had a greening rate of about 40% and at least five years of occupancy. For each habitat type, we selected four study sites, each separated by at least 1 km. According to the size of the park or residential area, we set up 1–3 sample lines (each about 1 km long) for the survey in each site. From March to May in 2021 and 2022, we conducted a survey once per month. The survey was carried out during the hours of 06:00–10:00 a.m. or 15:00–18:00 p.m. (the daily peaks of bird activity) on rainless days, and the observers walked at a speed of about 0.5–1.0 km/h and an observation distance of 50 m between the left and right of the sample line. We used binoculars and cameras to observe and record individual characteristics and behaviors, with at least two observers for each survey.

We recorded the songs of male oriental magpie-robins at each study site at 06:00–10:00 a.m. on rainless days during the peak of courtship (April 2022). To avoid recording the same individual twice, we visited each site only once, and the locations of the recorded birds were spaced at least 100 m apart. According to our observation, each oriental magpie-robin had a fixed singing perch during the breeding season, and rarely shared the perch. We searched for the target individuals by listening to the calls and observing the behavior of the adult birds. Only territorial songs (i.e., continuous polysyllabic songs) were recorded, with no other vocalizations (such as alarm calls or contact calls, which are often monosyllabic or disyllabic). Songs were recorded using a digital recorder (Marantz, PMD661, Fukushima, Japan) and a directional microphone (Sennheiser, ME66, Weidmark, Germany) pointed directly towards the singing individual. To reduce human interference, we kept a distance of 5–10 m from the target individuals. We recorded at least 10 complete songs for each individual, or until the target individual stopped singing or flew away. Recordings were sampled at 44.1 kHz, 16-bit resolution, mono, WAV format. We also recorded the singing perch type (i.e., artificial building or tree), the singing height of each individual, the background noise level of the sample site using a noise meter (deli, DL333202, Nibo, China), and the intensity of human activity classified according to three levels (high, medium, and low).

### 2.3. Song Analysis

**Figure 2:**
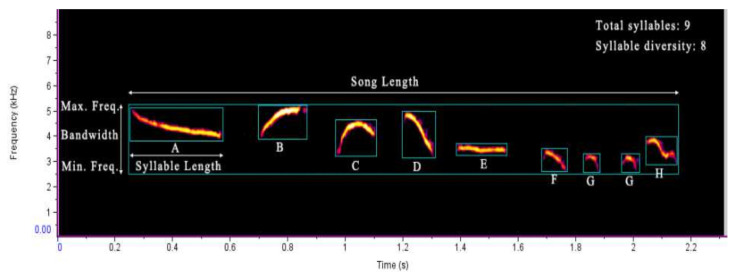
An example of the territorial song of the oriental magpie-robin and measurement of the song variables in the spectrogram. A–H stands for different syllable types.

Since we did not obtain valid male songs in high-rise residential areas, we only analyzed the songs recorded in the other two habitat types. Firstly, we used Raven Pro (version 1.6, Raven Team, Ithaca, NY, USA) to generate the spectrogram and sonogram of each song, with a 512-point fast Fourier transform (FFT) and an overlapping 50% Hamming window. Then, we selected the 10 highest-quality songs for each individual for further analysis after a visual inspection of each sonogram. Finally, we measured the following song characteristics from the selected spectrogram: song length (s), syllable number, syllable diversity (i.e., number of different syllable types per song, classified according to syllable structure), syllable length (s), maximum frequency (Hz), minimum frequency (Hz), and frequency bandwidth (Hz). We manually measured the characteristics of each song by using the precise placement of the selection boxes in the spectrogram view, and these data were automatically extracted and recorded by Raven (Figure 2). 

### 2.4. Statistical Analysis

The differences in the population density of the oriental magpie-robins between the three habitat types, along with the differences in song characteristics between the low-rise residential areas and the urban parks, were examined using linear mixed models, with “habitat type” as a fixed factor and “sample site” or “individual identity” as a random factor; Tukey’s HSD test followed if a significant difference was found. To meet the assumptions of normality and homogeneity of variance, the numerical data were transformed prior to statistical analysis, if necessary. The differences in the singing perch types of the oriental magpie-robins between low-rise residential areas and urban parks were examined using Fisher’s exact test. Principal component analysis (PCA) was used to assess the song similarity of each individual in different habitats, incorporating temporal and frequency variables (as mentioned above). Each individual was plotted as a spatial point created by the first two principal components. To explore the effects of the singing habitat variables on song characteristics, we also constructed linear mixed models using each of the song characteristics as the dependent factor and the habitat variables (i.e., habitat type, singing perch type, singing height, noise level, and human activity) as the independent fixed-effect factors, with “individual identity” and “sampling site” as the random factors. All statistical analyses and graphs were performed with R [22], using the packages ggplot2 [23] and lmerTest [24].

## 3. Results

**Figure 3:**
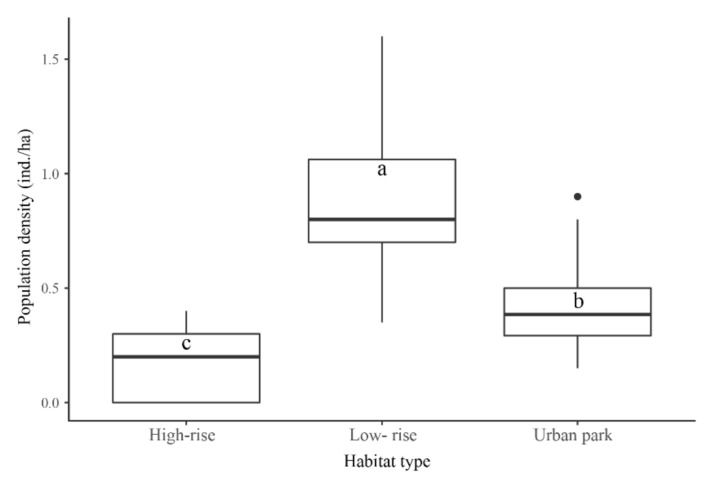
Differences in population density of the oriental magpie-robin between the three habitat types (*n* = 24; different letters a–c indicate significant differences between habitat types, *p* < 0.05).

We recorded the distribution of the oriental magpie-robin in all three habitat types (Figure 1d–f), but the population density varied significantly between habitat types (*F*_2, 9_ = 13.23, *p* < 0.01, Figure 3). The population density of the oriental magpie-robin in the low-rise residential areas (0.89 ± 0.06 ind./ha) was significantly higher than that in urban parks (0.42 ± 0.04 ind./ha), while the lowest population density was in high-rise residential areas (0.19 ± 0.03 ind./ha). 

We analyzed a total of 360 songs from 36 individuals; 230 songs belonged to 23 individuals in low-rise residential areas, while 130 songs belonged to 13 individuals in urban parks. Naturally, the difference in population distribution between the two habitats resulted in uneven song sampling, so the effective singing individuals that we recorded in urban parks were relatively limited. We found that in low-rise residential areas male oriental magpie-robins produced songs with a greater song length; a higher syllable number, maximum frequency, and bandwidth; and a lower minimum frequency than those in urban parks (Table 1). Male songs in low-rise residential areas had about three more syllables on average than those in urban parks (10.24 ± 0.52 vs. 7.20 ± 0.45, Table 1).

**Figure 4:**
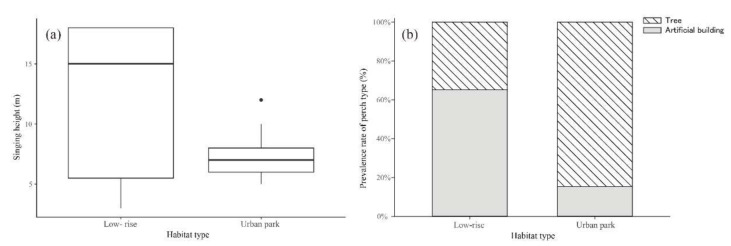
Differences in singing height (**a**) and perch type (**b**) of the oriental magpie-robins between low-rise residential areas and urban parks.

The singing height of oriental magpie-robins in low-rise residential areas (11.83 ± 1.32 m) was significantly higher than that in urban parks (7.46 ± 0.60 m) (*t* = 2.39, *p* < 0.05, Figure 4a). The selection of the singing perch by males in the two types of habitat also differed greatly (Fisher’s exact test, *p* < 0.05). Most male oriental magpie-robins (65.2%; 15 of the total 23 individuals) chose an artificial building as the singing perch in low-rise residential areas, while 34.8% of males chose a tree as the singing perch. However, in urban parks we recorded only two males using an artificial building, while 84.6% of males (11 of the total 13 individuals) sang in a tree (Figure 4b). This may be mainly because urban parks have fewer buildings and more trees than low-rise residential areas. There were no significant differences in the background noise level (51.59 ± 2.80 dB vs. 53.49 ± 1.62 dB, *t* = −0.63, *p* = 0.53) or the intensity of human activity (*z* = 1.39, *p* = 0.17) between urban parks and low-rise residential areas. This may be because there were fewer human activities during our recording period (06:00–10:00 a.m.). 

**Figure 5:**
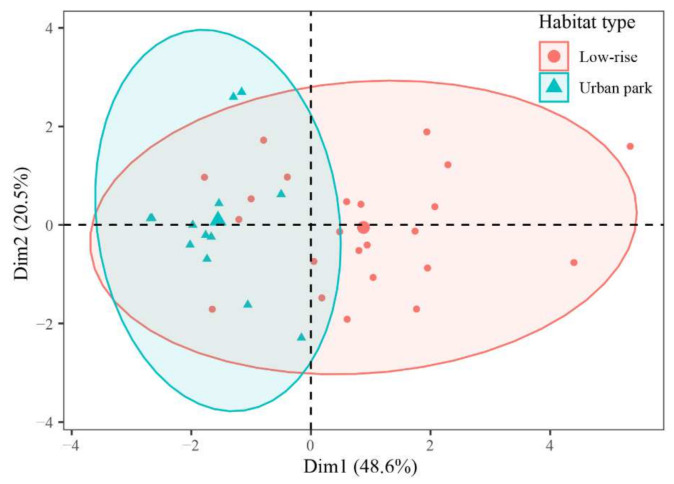
Plot of the PCA of song similarity of individuals between low-rise residential areas and urban parks by the first 2 PCA axes.

As shown in the PCA results, the first two principal components accounted for a total of 69.1% of the song variation between habitats; specifically, PC1 explained 48.6% of the variation, while PC2 explained 20.5% of the variation (Figure 5). The points representing the individual songs in the low-rise residential areas were more widely scattered, and covered a larger area of the coordinate axis, while the points representing those in urban parks were more concentrated, and covered a smaller area of the coordinate axis. These results indicated that the male oriental magpie-robins in low-rise residential areas had a higher diversity of songs.

According to the linear mixed model results, the habitat type significantly predicted the modifications of song length, syllable number, minimum frequency, and bandwidth in individual songs (Table 2). However, the singing height, perch type, noise level, and human activity did not seem to have significant effects on the variation in song characteristics (Table 2).

## 4. Discussion

Urbanization is a primary driver of habitat loss and fragmentation for most animals, but it also provides more opportunities for some urban dwellers [25], e.g., richer food resources or safer shelters [26,27]. According to our results, the oriental magpie-robin was found in all three urban habitat types in our survey area; thus, it should clearly be classified as an urban dweller. However, the population density of the three habitat types showed significant differences. As we expected, high-rise residential areas were the least popular, with an extremely low population density of oriental magpie-robins; hence, this emerging habitat type may also be unfamiliar and unavailable to urban dwellers. Interestingly, the population density was significantly higher in low-rise residential areas than in urban parks. This suggests that the oriental magpie-robin does like to be around human residential environments, but simply rejects high-rise buildings. A study in Sweden also showed that the density of great tit breeding pairs was highest in residential areas [28]. Oriental magpie-robins nest in tree cavities and non-natural nesting substrates, e.g., nest boxes or artificial buildings [29]. In our study, we observed that this bird mostly nested in the cavities of buildings in low-rise residential areas. It has been proven in some birds that nesting in artificial buildings can provide better benefits [30,31]. In addition, we also observed a certain number of oriental magpie-robins foraging near garbage cans in low-rise residential areas (Figure S1), where food resources may be more abundant than in urban parks. These factors may contribute to the high population density of oriental magpie-robins in low-rise residential areas. Of course, our results may be limited by the small sample size, and there is a need for a larger samples and long-term data for further verification.

There is a broad consensus on anthropogenic-environment-induced variation in birdsong [32], and many studies suggest that contextual differences may lead to changes in the frequency, length, and diversity of birdsong [13,33]. According to our results, the songs of the male oriental magpie-robin in low-rise residential areas had a greater song length; a higher syllable number, maximum frequency, and bandwidth; and a lower minimum frequency than those in urban parks. Male songs in low-rise residential areas had three more syllables than those in urban parks, and the average syllable length of the two habitats was not different, leading to a longer song length in low-level residential areas. Previous studies in South and South-East Asia showed that male oriental magpie-robin songs were about 0.8 s–2.8 s in length, consisted of 3–10 syllables, and had a frequency range of about 2000 Hz–6000 Hz [19,34,35]. These findings were generally consistent with our results. However, we found that many oriental magpie-robin songs in low-rise residential areas were much longer than 10 syllables, and some even consisted of more than 20 syllables (Figure S2). Some studies of other species have suggested that females consistently prefer males with longer songs, as this usually means higher body quality [36]. There may be more intense mating competition in low-rise residential areas due to the higher population density than in urban parks. This may lead to an increase in male song length and complexity. The PCA results also showed that male oriental magpie-robins in low-rise residential areas had a higher diversity of songs. Due to the founder effect, the song diversity of songbirds living on islands or in small populations is sometimes lower than that on the mainland or in large populations [37,38]. Larger population sizes in low-rise residential areas may provide the potential for the evolution of song complexity. The risk of predation may also limit the song length of male songbirds due to increased exposure [39]. According to our observations, low-rise residential areas are deficient in specialized avian predators, except for some cats, while predators in urban parks are more diverse, e.g., raptors and snakes. A lower risk of predation in residential areas may allow males to sing more comfortably for longer.

Some studies have suggested that differences in habitat type can lead to variation in birds’ singing height and post sites [40,41,42]. According to our results, oriental magpie-robins in low-rise residential areas sang at a higher position and made more use of artificial buildings than those in urban parks. Male birds always tend to choose the best singing perch to facilitate the transmission of acoustic signal, and a high and open perch is the usual choice when no other factors interfere [10]. Considering the landscape differences between the two habitats, it is clear that the roofs of buildings were the ideal singing perches for most males in low-rise residential areas, while trees were preferred in urban parks. However, the model results indicated that the most important predictive variable of song differences was habitat type, independent of singing height and perch type. In other words, oriental magpie-robins in low-rise residential areas had a greater song length and a higher syllable number and bandwidth than those in urban parks, regardless of singing height or perch type variation. Therefore, low-rise residential areas may provide preferred song post sites for most oriental magpie-robins in urban environments. Background noise levels and human activities are often considered to be important factors contributing to variation in birdsong [10,12]. A study in the Pearl River Delta region of China also showed that oriental magpie-robins sang at higher frequencies in noisier areas [21]. However, no effect of these two factors on the song characteristics of the oriental magpie-robin was found in our study. This may be due to the lower noise levels and lower intensity of human activity during the period that we recorded, resulting in little variation between habitats. In addition, this urban resident bird is very bold and active, and has a strong tolerance to general human activities.

The differences in the sound transmission properties of distinct habitats often affect birdsong divergence [43,44]. The acoustic signals of birds in open environments are less affected by attenuation and reverberation, so the propagation distance is longer than in a closed environment [10]. Compared to the woods of urban parks, the rooftops of low-rise residential areas should be a more open environment to oriental magpie-robins for singing. The architecture of high-rise residential areas is clearly not conducive to birdsong transmission. The trees in the natural habitat of the oriental magpie-robin do not exceed 20 m in height, and this bird is not capable of flying to the tops of high-rise buildings. When males sing in the middle of the floors, the blocking of the acoustic signal by the high-rise buildings significantly increases the attenuation. In future studies, the sound transmission properties in the three habitat types should be tested for better understanding of the variation in the songs of oriental magpie-robins.

## 5. Conclusions

In summary, the findings of our study showed that most oriental magpie-robins living in urban areas prefer low-rise residential areas as habitats over urban parks, while they were rare in high-rise residential areas. Meanwhile, males in low-rise residential areas had a greater song length, syllable number, frequency bandwidth, and song diversity than those in urban parks. The song differences were mainly related to habitat types, independent of singing height and perch type. Our study suggests that the urban-dwelling oriental magpie-robin is well-adapted to the common low-rise building morphology, but shows a rejection of the emerging high-rise buildings. We recommend expansive studies on the effects of the variation in urban morphology on resident animals to determine which urban morphologies are conducive to enhancing biodiversity and encouraging animals to settle in urban areas.

## Figures and Tables

**Table 1:** Song characteristics of oriental magpie-robins analyzed between low-rise residential areas and urban parks (data are shown as the mean ± SE).

Song Characteristics	Habitat Types		*df*	*F*	*p*
	Low-Rise Residential Area (*n* = 23)	Urban Park (*n* = 13)			
Song length (s)	2.63 ± 0.18	1.61 ± 0.06	1, 34	16.86	<0.001
Syllable number	10.24 ± 0.52	7.20 ± 0.45	1, 34	15.61	<0.001
Syllable diversity	6.84 ± 0.33	6.20 ± 0.44	1, 34	1.34	0.26
Syllable length (s)	0.18 ± 0.01	0.15 ± 0.01	1, 34	1.41	0.24
Minimum frequency (Hz)	2266.27 ± 65.06	2618.81 ± 66.02	1, 34	12.44	<0.01
Maximum frequency (Hz)	6031.86 ± 134.18	5551.50 ± 147.94	1, 34	5.20	0.03
Bandwidth (Hz)	3773.03 ± 160.00	2932.68 ± 128.24	1, 34	12.85	<0.01

**Table 2:** Summary of linear mixed-effects models in testing the impacts of habitat type, singing perch, sing height, noise level, and human activity on song characteristics (* *p* < 0.05).

Song Characteristics	Variables	Estimate	*df*	*t*	*p*
Song length	Habitat type	0.765	31	2.311	0.028 *
	Singing perch	0.039	31	0.067	0.947
	Singing height	0.089	31	0.118	0.907
	Noise level	−0.280	31	−0.269	0.789
	Human activity	0.205	31	0.250	0.804
Syllable number	Habitat type	2.242	31	2.168	0.038 *
	Singing perch	1.800	31	1.005	0.322
	Singing height	−0.108	31	−0.046	0.964
	Noise level	1.833	31	0.913	0.368
	Human activity	1.583	31	0.645	0.523
Syllable diversity	Habitat type	0.713	31	0.955	0.347
	Singing perch	−0.988	31	−0.764	0.450
	Singing height	0.975	31	0.573	0.571
	Noise level	1.200	31	1.009	0.320
	Human activity	−0.400	31	−0.269	0.790
Syllable length	Habitat type	0.036	31	1.318	0.197
	Singing perch	−0.067	31	−1.404	0.170
	Singing height	0.003	31	0.054	0.957
	Noise level	−0.022	31	−0.320	0.751
	Human activity	0.006	31	0.112	0.912
Minimum frequency	Habitat type	−369.45	31	−2.715	0.011 *
	Singing perch	158.23	31	0.671	0.507
	Singing height	−131.29	31	−0.423	0.675
	Noise level	−192.60	31	−0.814	0.422
	Human activity	−55.44	31	−0.177	0.861
Maximum frequency	Habitat type	346.94	31	1.213	0.234
	Singing perch	14.37	31	0.029	0.977
	Singing height	−443.07	31	−0.679	0.502
	Noise level	−573.9	31	−1.434	0.161
	Human activity	439.9	31	1.564	0.128
Bandwidth	Habitat type	716.4	31	2.249	0.032 *
	Singing perch	−143.8	31	−0.261	0.796
	Singing height	−311.8	31	0.429	0.671
	Noise level	−451.3	31	−0.738	0.466
	Human activity	595.4	31	1.284	0.208

## Data Availability

The data that support the findings of this study are available from the corresponding author upon reasonable request.

## Supplementary Materials

The following supporting information can be downloaded at: https://www.mdpi.com/article/10.3390/ani12182436/s1, Figure S1: A male oriental magpie-robin foraging on a garbage can in a low-rise residential area; Figure S2: A territorial song of the oriental magpie-robin, consisting of 26 syllables in the spectrogram.
