# Supplementary figures and images for: Urban Low-Rise Residential Areas Provide Preferred Song Post Sites for a Resident Songbird
Source: Animals (Basel). 2022 Sep 15;12(18):2436. doi: 10.3390/ani12182436 (PMC9494978; doi:10.3390/ani12182436)

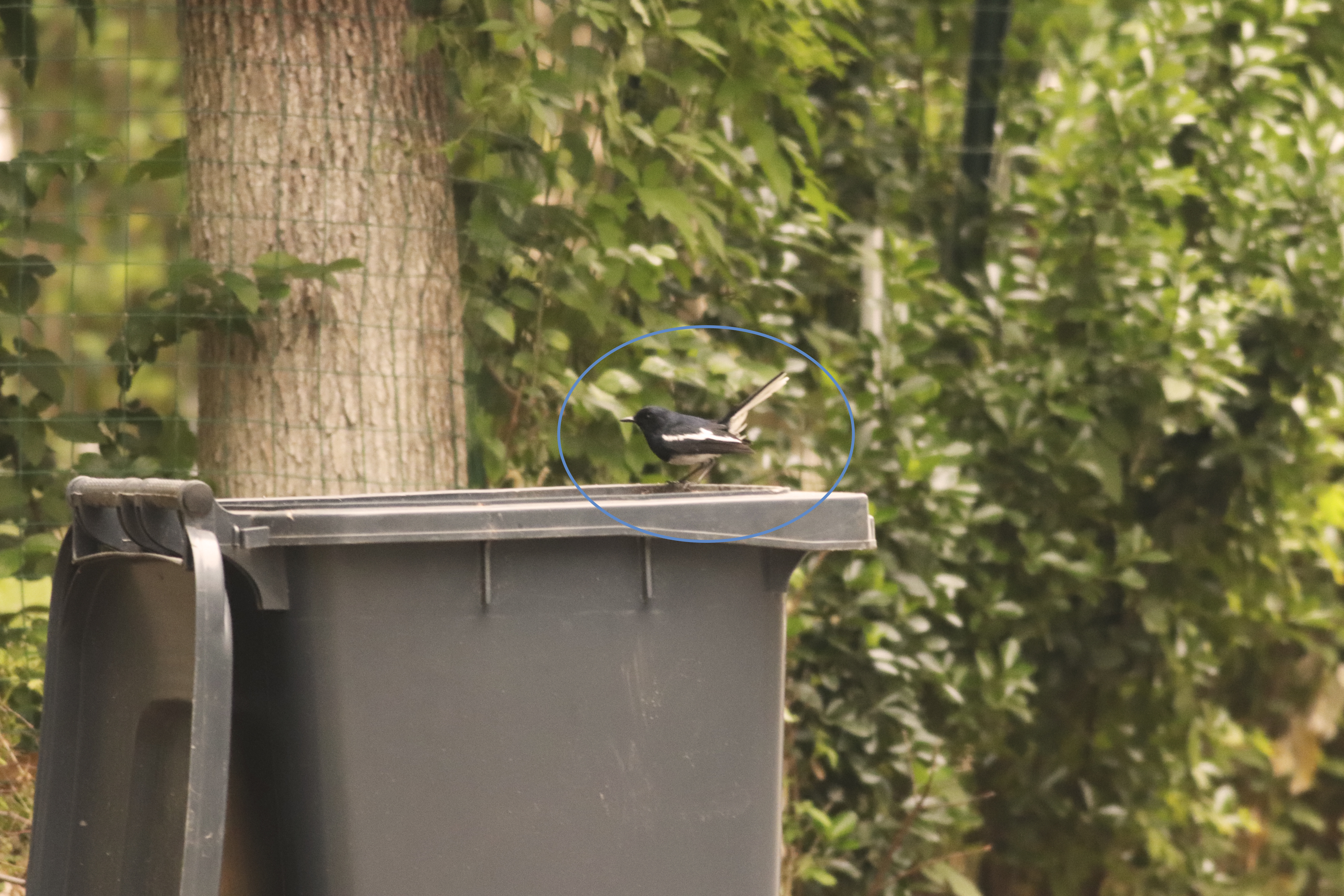

Supplement: Supplementary file 1 [file animals-12-02436-s001.zip › Fig. S1 and S2/Fig. S1 .JPG]

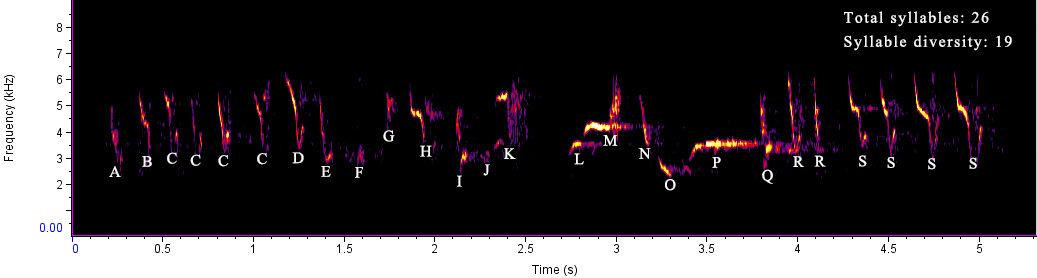

Supplement: Supplementary file 1 [file animals-12-02436-s001.zip › Fig. S1 and S2/Fig. S2 .jpg]
